# Pre-Harvest Survival and Post-Harvest Chlorine Tolerance of Enterohemorrhagic *Escherichia coli* on Lettuce

**DOI:** 10.3390/toxins11110675

**Published:** 2019-11-19

**Authors:** Deepti Tyagi, Autumn L. Kraft, Sara Levadney Smith, Sherry E. Roof, Julie S. Sherwood, Martin Wiedmann, Teresa M. Bergholz

**Affiliations:** 1 Department of Microbiological Sciences, North Dakota State University, Fargo, ND 58102, USA; kkdeeptityagi@gmail.com (D.T.); autumn.l.kraft@ndsu.edu (A.L.K.); Sara.levadney@ndsu.edu (S.L.S.); j.sherwood@ndsu.edu (J.S.S.); 2 Department of Food Science, Cornell University, Ithaca, NY 14853, USA; Ser15@cornell.edu (S.E.R.); mw16@cornell.edu (M.W.)

**Keywords:** EHEC, pre-harvest, survival, chlorine wash, lettuce, transcriptome

## Abstract

In the field, foodborne pathogens such as enterohemorrhagic *Escherichia coli* (EHEC) are capable of surviving on produce over time, yet little is known about how these pathogens adapt to this environment. To assess the impact of pre-harvest environmental conditions on EHEC survival, we quantified survival on romaine lettuce under two relative humidity (75% and 45%) and seasonal conditions (March and June). Greenhouse-grown lettuce was spray-inoculated with EHEC and placed in a growth chamber, mimicking conditions typical for June and March in Salinas Valley, California. Bacteria were enumerated on days 0, 1, 3, and 5 post-inoculation. Overall, we found that the effect of relative humidity on EHEC survival depended on the seasonal conditions. Under June seasonal conditions, higher relative humidity led to lower survival, and lower relative humidity led to greater survival, five days post-inoculation. Under March seasonal conditions, the impact of relative humidity on EHEC survival was minimal over the five days. The bacteria were also tested for their ability to survive a chlorine decontamination wash. Inoculated lettuce was incubated under the June 75% relative humidity conditions and then washed with a 50 ppm sodium hypochlorite solution (40 ppm free chlorine). When incubated under June seasonal conditions for three to five days, EHEC strains showed increased tolerance to chlorine (adj. *p* < 0.05) compared to chlorine tolerance upon inoculation onto lettuce. This indicated that longer incubation on lettuce led to greater EHEC survival upon exposure to chlorine. Subsequent transcriptome analysis identified the upregulation of osmotic and oxidative stress response genes by EHEC after three and five days of incubation on pre-harvest lettuce. Assessing the physiological changes in EHEC that occur during association with pre-harvest lettuce is important for understanding how changing tolerance to post-harvest control measures may occur.

## 1. Introduction

In recent years, an increased number of gastroenteritis outbreaks have been linked to fresh fruits and vegetables. Enterohemorrhagic *Escherichia coli* (EHEC) is ranked among the top five foodborne pathogens with the highest rates of hospitalization [1], and a number of outbreaks associated with this pathogen have been attributed to consumption of fresh produce [2,3]. For example, outbreaks of EHEC have been linked to consumption of alfalfa sprouts [4] and raw clover sprouts [5]. The Centers for Disease Control and Prevention estimated that produce accounted for almost half of all foodborne illnesses (46%) from 1998 to 2008, and of those illnesses linked to produce, 22% were attributed to leafy greens [6]. Among leafy green vegetables, spinach and lettuce have been most frequently implicated in outbreaks with enteric pathogens [7,8,9,10,11,12].

The transmission of enteric pathogens to fresh produce is complex and can occur in the pre-harvest environment via a number of different routes. Usage of contaminated irrigation water, application of raw manure, direct fecal deposition by wildlife, and improper worker hygiene are a few ways in which the produce could become contaminated in the field [13]. Once these pathogens are deposited on produce, they are capable of surviving in this non-host environment for lengthy periods of time [14,15,16,17]. The extent of their survival on pre-harvest produce can be significantly affected by environmental factors encountered in the field. The impact of specific pre-harvest stresses, such as humidity and UV exposure, on the survival of enteric pathogens have been assessed. For example, *Salmonella* Typhimurium ATCC 43,971 survived better on lettuce under high humidity as compared to low humidity [18]. In an experiment by Erickson et al., EHEC O157:H7 inoculated on the lower side of the lettuce leaf survived longer than pathogens inoculated on the upper side of the leaf [14]. Biological factors such as the age of the leaf and the life stage of the plant have also been found to influence survival of enteric pathogens on lettuce [19,20].

In addition to environmental stress in the field, pathogens are also exposed to a variety of stresses in the post-harvest environment. For lettuce and other leafy greens, this typically includes a decontamination wash. Chlorine, in the form of sodium hypochlorite, is commonly used as a sanitizer in the fresh produce industry [21,22]. Many studies have focused on evaluating the efficacy of chlorine-based decontamination methods to reduce EHEC on lettuce and other fresh produce [23,24,25,26]. These studies have typically inoculated post-harvest produce with bacterial cultures grown under optimal laboratory conditions, which does not reflect conditions that pathogens experience in the pre-harvest environment. It is important to assess if pathogen exposure to stresses in the pre-harvest environment can impact subsequent survival during post-harvest sanitizing, which would have implications for the efficacy of sanitizing treatments.

Once pathogens are present on produce, stresses present in the pre-harvest environment have the potential to influence their survival. Adaptation to the pre-harvest stresses may potentially lead to cross-protection against subsequent stresses, such as those experienced during post-harvest processing. The goals of this study were to determine how seasonal conditions and relative humidity (RH) influenced the survival of EHEC on lettuce, if the association of this pathogen with pre-harvest lettuce influenced subsequent survival in a chlorine wash, and how EHEC responded to conditions on pre-harvest lettuce over time.

## 2. Results

**Figure 1:**
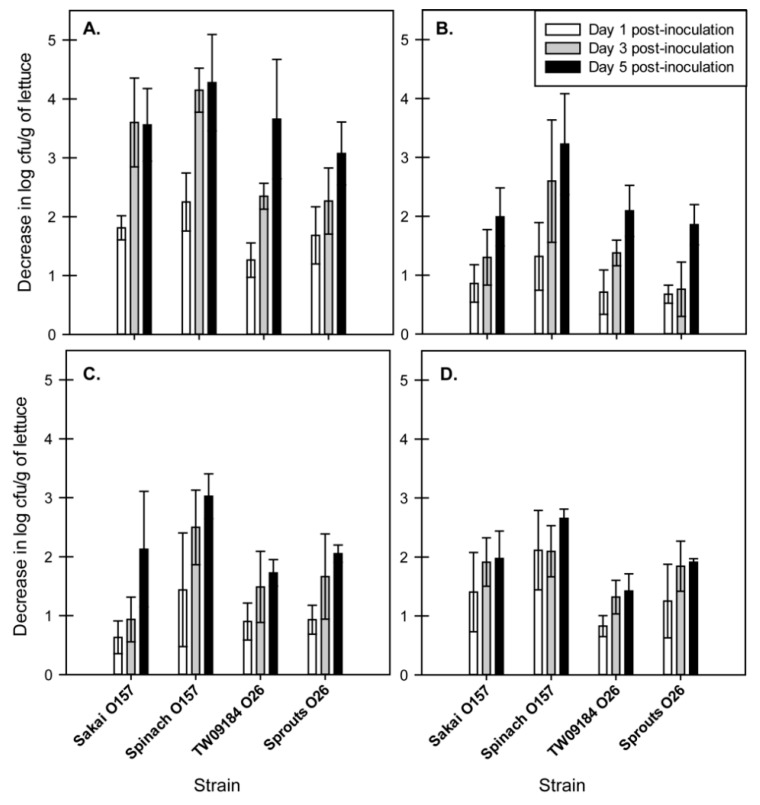
Bar graphs representing log decrease in survival of EHEC on lettuce over 5 days under June 75% RH (**A**), June 45% RH (**B**), March 75% RH (**C**), and March 45% RH (**D**). Bars represent the average and standard deviation from two independent replicates and two technical replicates for each strain.

We enumerated viable cells of four strains of EHEC (Table 1) on lettuce on days 0, 1, 3, and 5 post-inoculation to determine the effect of two seasonal (June and March) and RH conditions (45% and 75%) (Table 2). As the initial concentrations of each strain varied on the lettuce plants, the log decrease in CFU/g of lettuce was calculated for each day post-inoculation and used for comparison of survival across the different environmental conditions (Figure 1). All EHEC strains showed a decrease in cell number over five days, regardless of season or RH.

### 2.1. Effect of RH on EHEC Survival is Dependent on the Seasonal Conditions

RH had a significant effect on the survival of EHEC strains, which was dependent on the seasonal conditions (*p* < 0.0001) (Figure 1). Under June seasonal conditions, higher RH led to significantly lower survival of EHEC, while lower RH led to significantly greater survival. One day post-inoculation, an average decrease of 1.8 ± 0.5 log CFU/g of lettuce was observed under 75% RH (Figure 1A), whereas an average decrease of 0.9 ± 0.4 log CFU/g of lettuce was observed under 45% RH (Figure 1B). Three days post-inoculation, an average decrease of 3.1 ± 0.9 log CFU/g of lettuce was observed under 75% RH, whereas an average decrease of 1.5 ± 0.8 log CFU/g of lettuce was observed under 45% RH. Five days post-inoculation, an average decrease of 3.6 ± 0.8 log CFU/g of lettuce was observed under 75% RH, and an average decrease of 2.3 ± 0.8 log CFU/g was observed under 45% RH.

The total impact of RH on EHEC survival was minimal over the five days for the March seasonal conditions. One day post-inoculation, an average decrease of 1.0 ± 0.6 log CFU/g of lettuce under 75% RH (Figure 1C), and an average decrease of 1.4 ± 0.7 log CFU/g of lettuce under 45% RH (Figure 1D) was observed. Three days post-inoculation, an average decrease of 1.6 ± 0.8 log CFU/g of lettuce under 75% RH, and an average decrease of 1.8 ± 0.5 log CFU/g of lettuce was observed under March 45% RH. Five days post-inoculation, an average decrease of 2.2 ± 0.7 log CFU/g and 2.0 ± 0.5 log CFU/g of lettuce was observed for 75% and 45% RH, respectively.

### 2.2. EHEC Strains Demonstrated Changes in Tolerance to Chlorine over Time

Each strain was tested for the ability to survive a decontamination wash with chlorine (sodium hypochlorite) after inoculation onto lettuce on day 0 and after being exposed to June 75% RH on lettuce plants 1, 3, and 5 days post-inoculation. June 75% RH incubation conditions were selected based on the survival experiments, indicating this condition led to the overall greatest decrease in pathogen populations on lettuce (Figure 1A). As some bacterial cells may be removed from the lettuce surface due to the agitation during washing, the log CFU/g lettuce obtained from lettuce washed with chlorine was compared to that recovered from lettuce washed with buffered water. These data are displayed as the difference in recovery between buffered water wash and chlorine wash (defined as “reduction attributable to chlorine”). EHEC strains inoculated onto lettuce before incubation under June 75% RH conditions (day 0) varied in the reduction of cells attributable to chlorine, with O26 TW09184 having the greatest average reduction of 1.13 ± 0.06, significantly higher (adj. *p* < 0.05) than that of O157 spinach (0.67 ± 0.10 log CFU/g) and O26 sprouts (0.64 ± 0.26 log CFU/g) (Table 3). Significant differences in reduction attributable to chlorine were not observed among the four strains on days 1, 3, or 5 post-inoculation (Table 3). When comparing the reduction attributable to chlorine over the five days for each strain, we found that both O157 strains had significantly (adj. *p* < 0.05) lower reduction on days 3 and 5 compared to day 0, and both O26 strains had significantly (adj. *p* < 0.05) lower reduction on day 3 compared to day 0 (Table 3). This indicates that longer incubation on lettuce led to greater survival after the chlorine wash for these EHEC strains, though the duration of this decreased sensitivity to chlorine varied by strain.

### 2.3. Changes in EHEC Transcriptomes During Incubation on Lettuce Plants

Strains O157 Sakai and O26 sprouts were selected for transcriptome analysis. Global gene expression of each strain was determined on days 1, 3, and 5 post-inoculation, and significant changes in gene expression over time were identified. There was a total of 90 O157 Sakai genes and 180 O26 sprouts genes that were significantly upregulated on at least one day compared to the other time points (Tables S1 and S2). Only five genes were upregulated in both strains: *bssS,* ECs_2291, *elaA, lacY,* and ECs_4188. Expression of these genes was significantly higher on day 5 compared to either day 1 or day 3 in O157 Sakai, while expression was significantly higher on days 3 and 5 compared to day 1 in the O26 sprouts strain.

#### 2.3.1. Changes in O157 Sakai Gene Expression During Incubation on Lettuce Plants

The majority of significant changes in gene expression occurred on day 5 for O157 Sakai, with greater transcript levels on day 5 compared to both day 1 and day 3 (Table 4). A subset of those genes also had higher transcript levels on day 3 compared to day 1. Genes encoding proteins involved in attachment and biofilm formation (*bhsA, bssS*) had 3- to 4-fold greater transcript levels on day 5 compared to either day 1 or day 3. Genes encoding proteins with roles in stress resistance were also upregulated on day 5, including *gadE* and *uspB.* Seven genes encoding type 3 secretion system effectors were significantly upregulated on day 5, along with five genes encoding type 3 secretion system proteins. The majority of significantly differentially expressed genes (46/90) were annotated as hypothetical proteins (Table S1).

#### 2.3.2. Changes in O26 Sprouts Strain Gene Expression During Incubation on Lettuce Plants

The majority of significant changes in gene expression occurred on days 3 and 5 for the O26 sprouts strain, with greater transcript levels on days 3 and 5 compared to day 1 (Table 5 and Table S2). No genes were identified with significantly higher transcript levels on day 1 compared to either day 3 or day 5. Genes encoding molecular chaperones, including *dnaK, grpE, groES,* and *hslUV,* were significantly upregulated on days 3 and 5. Genes encoding proteins involved in the general stress response were significantly upregulated on days 3 and 5, including *rpoS, ostAB, tktB, glgS, yeaG, ycgB, yciEF, osmC, sodC,* and *hdeA.* Two of these RpoS-regulated genes, *osmC* and *sodC,* encode proteins with roles in managing oxidative stress. OxyR-regulated genes *yaiA* and *mntH* were also upregulated on days 3 and 5. Activation of these oxidative stress response genes may contribute to the decreased sensitivity to chlorine observed on day 3 for the O26 sprouts strain (Table 3).

## 3. Discussion

### 3.1. Pathogen Survival on Pre-Harvest Lettuce under Different RH is Dependent on Seasonal Conditions

We quantified survival of EHEC over a five-day period on romaine lettuce under two RH and seasonal conditions and demonstrated that the effect of RH on survival was dependent on the seasonal conditions. A previous study by Stine et al. compared survival of single strains of EHEC O157:H7 and *S.* Typhimurium under low RH (~45%) and high RH (~90%) on pre-harvest cantaloupe, lettuce, and peppers when light, CO_2_, and temperature remained constant [18]. They found that EHEC O157 had higher survival on cantaloupe under high RH, while, consistent with our data, survival was greater on lettuce under low RH. For the EHEC isolates we tested, RH significantly impacted survival under June seasonal conditions leading to lower survival under high RH and greater survival under lower RH over five days. However, the effect of RH on EHEC survival was minimal under March seasonal conditions. The two main differences between June and March seasonal conditions that we used are a longer photoperiod (14.8 h for June, 12 h for March) and higher maximum and minimum temperatures (20 °C and 12.2 °C for June, 17.2 °C and 6.7 °C for March).

A longer photoperiod would presumably lead to greater UV exposure, which is considered a major stressor for microbes on the leaf surface [27]. A study by Erickson et al. revealed that EHEC O157 sprayed on the lower side of leaves of field-grown lettuce resulted in greater survival of the pathogen than those sprayed on the upper side of leaves, demonstrating the role of increased UV exposure in reduced survival of EHEC on lettuce [14]. Tomas-Callejas et al. reported a reduced rate of inactivation for EHEC O157 on three different types of baby leafy greens under fall season growth conditions compared to summer, likely due to reduced daylight exposure, as temperature and RH were held at similar levels across seasons [28]. Overall, our data, along with previous studies, suggest that survival of EHEC on produce is affected by a combination of factors, including, but not limited to, RH, temperature, and UV exposure.

Enteric pathogens have the capability of responding to adverse environments by inducing a state of dormancy or inactiveness, referred to as the viable but non-culturable (VBNC) state [29,30]. Pathogens in a VBNC state fail to appear on routine culture media but remain viable for long time periods while maintaining virulence properties [31,32]. EHEC O157:H7 has been demonstrated to enter the VBNC state when associated with lettuce [33]. As we used colony counts on selective media to quantify survival, injured and/or VBNC cells would not be measured; therefore, apparent differences in die-off may also represent differences in the proportion of cells that enter a VNBC state under the different conditions. Moyne et al. compared the number of viable EHEC O157:H7 on pre-harvest lettuce over time after inoculation using colony counts and Q-PCR, and found that number of culturable cells was significantly lower than the number of viable cells [34]. The specific environmental factor(s) that may induce this physiological state on pre-harvest produce are yet to be elucidated.

### 3.2. Association of EHEC with Pre-Harvest Lettuce Impacts Chlorine Tolerance

Chlorine is a widely used sanitizer in fresh produce wash water, and its efficacy at inactivating enteric pathogens on fresh produce as well as in wash water has been evaluated in a number of studies [22,35,36,37]. We evaluated the efficacy of chlorine to inactivate EHEC when the pathogen had been associated with pre-harvest lettuce over time by comparing the number of cells remaining on lettuce after washing with buffered water and with 50 ppm chlorine. On the day of inoculation, pathogen reduction due to chlorine ranged from 0.33 to 1.13 log CFU/g lettuce. These findings are similar to those reported in other studies, where washing lettuce or other leafy greens with chlorine-based compounds resulted in ~1 log reduction of EHEC [26,28]. Additionally, both O157 strains demonstrated a further increase in chlorine resistance as the length of time on the lettuce plant increased.

In our study, we also identified variability in chlorine tolerance among strains of EHEC. Differences in sanitizer tolerance among serotypes and/or genetic subtypes of foodborne pathogens have been reported for EHEC [38,39]. Shen et al. examined variation in chlorine tolerance in produce wash water among multiple serotypes of *Salmonella* and Shiga toxin-producing *E. coli* (STEC), and found, consistent with our day 0 findings, that among STEC, O157:H7 strains were more tolerant than the non-O157 STEC tested [39]. Deng et al. assessed multiple strains representing the ‘big 6′ non-O157 STEC serotypes as well as O157:H7 and found variation in chlorine sensitivity and transfer from inoculated lettuce leaves to wash water among the serotypes. A meta-analysis of fresh produce sanitizer treatments identified that EHEC O157:H7 seems to present intrinsically higher tolerance to the range of commonly used sanitizers compared to *Salmonella* and *L. monocytogenes* [40].

Chlorine tolerance can be influenced by a number of factors, including physical protection of the bacterium by features of the plant [41,42] and induction of stress responses by the bacterium. EHEC that are able to survive on pre-harvest lettuce could be adapting to stresses present in the phyllosphere, and through that adaptation, have the potential to induce cross-protection against subsequent stresses, such as oxidative stress in the form of chlorine. While not the same experimental design that we used, Kyle et al. exposed EHEC O157 to lettuce leaf lysate and observed the induction of genes involved in oxidative stress. Further testing showed that exposing the pathogen to lysate led to increased tolerance to chlorinated sanitizers [43].

### 3.3. Upregulation of Virulence Genes in O157 Sakai During Association with Pre-Harvest Lettuce

Transcriptome data from O157 Sakai indicates the upregulation of genes with roles in virulence in particular components of the type 3 secretion system (TTSS) and TTSS effectors. Increased expression of TTSS genes *eivE, escU,* and *escD* was previously observed for O157 Sakai after two days on pre-harvest lettuce [44], and 33 TTSS structural and effector genes were upregulated in O157 EDL 933 after 30 min exposure to lettuce leaf lysate [43]. While our experiment was not designed to identify which environmental cues may be leading to the upregulation of EHEC virulence genes, other researchers have demonstrated increased expression of TTSS genes in O157 Sakai exposed to osmotic stress [45]. Our data, as well as that from other O157 transcriptome experiments, suggest that EHEC could be experiencing osmotic stress when associated with plants [46,47].

### 3.4. Gene Expression Changes Indicate Activation of Stress Responses During Association with Pre-Harvest Lettuce

Transcriptome data from the O26 sprouts strain indicates the upregulation of genes contributing to osmotic stress resistance and oxidative stress resistance. Many of these genes are regulated by RpoS (*sodC, osmC, elaB*) [48,49] or OxyR (*yaiA, mntH*) [50], and elements of the oxidative stress response can be upregulated in response to osmotic stress [51]. Upregulation of these genes on day three post-inoculation may contribute to the increased chlorine resistance observed on day three for the O26 sprouts strain. Similar upregulation of RpoS regulated genes, including *otsAB, tktB,* and *sodC* was observed by Fink et al., for O157 EDL933 inoculated onto post-harvest lettuce for three days [46]. In contrast, Van der Linden et al. reported decreased expression of oxidative stress-related genes *soxS* and *sodA* in O157 Sakai after two days on lettuce plants [44]. The gene encoding BhsA, involved in biofilm formation and stress response (and formerly known as *ycfR*) [52], was upregulated in both O26 sprouts and O157 Sakai on pre-harvest lettuce. Similar upregulation was previously observed for O157 EDL 933 on post-harvest lettuce [46], O157 EDL 933 in lettuce lysate [43], and O157 Sakai on pre-harvest lettuce [44]. BhsA was found to contribute to the attachment of O157 EDL 933 to lettuce leaves [46] and appears to be a common response across different EHEC strains to the lettuce environment.

## 4. Conclusions

An assessment of the pre-harvest survival of EHEC on lettuce indicated an important role of environmental factors in pathogen survival. We found that pre-harvest environmental factors, such as the seasonal photoperiod and minimum and maximum temperature, as well as RH, impacted the survival of EHEC on lettuce. Our study also revealed that the chlorine tolerance of EHEC is impacted by association of the pathogen with lettuce and can increase over time, leading to increased survival after a post-harvest decontamination treatment. Transcriptome data revealed the upregulation of osmotic and oxidative stress response genes in EHEC O26 sprouts, indicating that adaptation of EHEC to environmental conditions on pre-harvest lettuce could be associated with increased tolerance to chlorine. These results highlight the importance of understanding pre-harvest stressors that may lead to increased tolerance of enteric pathogens to produce decontamination treatments.

## 5. Materials and Methods

### 5.1. Bacterial Isolates and Growth Conditions

Two isolates, each of EHEC serotypes, O157 and O26, were used in this study (Table 1). All isolates were stored at −80 °C in Brain-Heart Infusion (BHI) broth with 10% glycerol. Each bacterial isolate was freshly streaked to Luria-Bertani (LB) agar from frozen stock and incubated for 24 h at 37 °C. A single colony was transferred to 5 mL of LB and incubated at 37 °C for 15 h. After 15 h, 100 μL of LB culture was transferred to 100 mL of LB broth and incubated at 37 °C, 215 rpm, for 15 h.

### 5.2. Lettuce Cultivation Conditions

Romaine lettuce seeds (*Lactuca sativa*) purchased from Living Whole Foods (Springville, UT, USA) were seeded into soil (Sungro Sunshine LC1 consisting of coarse perlite, dolomitic limestone, gypsum, and Canadian sphagnum peat moss) in 4.5-inch plastic pots. Lettuce was grown in the North Dakota Agricultural Research Experiment Station greenhouse facility at 13–15 °C during the night and 18–20 °C during the day with a photoperiod of 14.5 h. The average RH in the greenhouse space was ~50%. Plants were watered as needed.

### 5.3. Preparation of Inoculum and Lettuce Inoculation

Following growth in LB for 15 h, bacterial cells were collected by centrifugation at 8000× *g* for 5 min (Avanti J-25 Centrifuge, Beckman Coulter, Brea, CA, USA). After discarding the supernatant, the inoculum was prepared by suspending the cell pellet in 50 mL phosphate-buffered saline (PBS) for a final concentration of approx. 10^9^ CFU/mL. After 28–35 days of growth, leaves were approximately 4 to 5 inches long, and 8 pots of lettuce were inoculated with each isolate via spray inoculation in a biosafety cabinet. A hand-held TLC sprayer (model 422530-0050, Kontes Glass Company, Vineland, NJ, USA) was used to deliver inoculum by spraying for 5 s (approx. 1 mL) from the top onto the lettuce leaves of each pot [21,22]. The carrier gas was nitrogen at approximately 10 Psi. Inoculated plants were placed in a plastic tray filled with 2 cm water and kept in a growth chamber (Conviron PGW40, Winnipeg, MB, Canada) approximately 5 feet from the light source. The photon flux in the growth chamber was 1400 µmoles/m^2^/s and is a measure of the quality of light for plant growth.

### 5.4. Incubation Conditions for Inoculated Lettuce

To quantify the impact of humidity and harvest season on pathogen survival, the inoculated lettuce plants were incubated under two RH levels (45% and 75%). Lettuce plants were incubated under two conditions mimicking two harvest seasons in Salinas Valley, California: June (14.8 h photoperiod, max temp 20 °C, min temp 12.2 °C) and March (12 h photoperiod, max temp 17. 2 °C, min temp 6.7 °C); for a total of 4 different environmental conditions (March 75% RH, March 45% RH, June 75% RH, June 45% RH). Climate data for these seasons were obtained from the Salinas Municipal Airport weather station for 2009–2011 from the National Climatic Data Center (http://www.ncdc.noaa.gov/). For each environmental condition, two independently grown cultures of each strain (biological replicates) were tested. For each biological replicate of each strain, plants from two lettuce pots were collected on each day of harvest. Each lettuce pot represents a technical replicate. One set of technical replicates were collected immediately after inoculation (day 0) while the rest were collected on days 1, 3, and 5 post-inoculation. Sterile scissors and forceps were used to cut lettuce leaves approximately one inch above the soil.

### 5.5. Incubation and Collection of Lettuce for RNA Isolation

For a subset of strains (Sakai and TW016501), additional sets of lettuce plants were inoculated as described above and incubated under the June 75% RH conditions. For each biological replicate of each strain, plants from four lettuce pots were collected on each day of harvest and combined into one sample for RNA isolation [53]. Lettuce was harvested on days 1, 3, and 5 post-inoculation. Harvested leaves were placed into a sterile filter bag, and 200 mL of physiological saline and 20 mL of ice-cold freshly prepared stop solution (10% acid phenol in ethanol) were added. Bags were sealed and kept on a rotator for 15 min at 200 rpm at 4 °C. Homogenate was collected into a 250 mL centrifuge bottle. To pellet cells, homogenate was immediately centrifuged at 10,000 rpm for 20 min at 4 °C. The supernatant was discarded, and the cell pellet was suspended in 2 mL lysis buffer (20 mM EDTA, 200 mM sodium chloride) and transferred to a bead-beating tube containing ~1 cc acid washed 0.1 mm zirconium beads. For cell lysis, 3 mL acid phenol, 0.1 mL 20% sodium dodecyl sulfate (SDS), and 100 mg polyvinylpolypyrrolidone (PVPP) were added and samples homogenized in a bead-beater (Biospec Products, Bartlesville, OK, USA) for 3 min. Supernatant was collected and hot acid phenol-chloroform was immediately added, and the tube was held at 65 °C for 1 h with periodic shaking. The supernatant was extracted with acid phenol-chloroform-isoamyl alcohol (125:24:1). RNA was precipitated in 2.5 volume of 100% ethanol, 1/10 volume 3 M sodium acetate, pH 5.2, and 1/100 volume glycogen overnight at −80 °C. The RNA sample was treated with DNase using DNase (Promega, Madison, WI, USA), RQ1 Buffer (Promega), and 0.1 M DTT to remove genomic DNA. Extracted RNA was quantified using a spectrophotometer (ND-1000), and the quality and integrity were analyzed using an Agilent 2100 bioanalyzer (Agilent Technologies, Santa Clara, CA, USA).

### 5.6. RNA Sequencing

The rRNA was depleted using Ribo-Zero rRNA removal kit (Epicentre Technologies, Chicago, IL, USA), and cDNA was synthesized using ScriptSeq complete kit (Epicentre Technologies) following the manufacturer’s instructions. cDNA libraries were barcoded using the Epicentre indexing primers to allow for multiple samples to be run in the same sequencing lane. Sequencing was performed on Illumina Hiseq at the Biotechnology Resource Center at Cornell University. Each flow cell consisted of 12 samples per lane to obtain 100 bp single-end sequencing reads. Sequencing reads for each strain and each sample are available at the NCBI SRA, PRJN A380789.

### 5.7. Genome Sequencing

Only one of the strains selected for RNA-seq analysis had a genome sequence available (O157:H7 Sakai, BA000007.3). DNA was isolated from TW16501 using a Qiagen DNeasy kit. Libraries were prepared from genomic DNA using TruSeq (Illumina, San Diego, CA, USA), and 250 bp paired end reads were sequenced on a MiSeq at the Cornell University Biotechnology Resource Center. Draft genomes were assembled de novo using Velvet [21,22]. Contigs for each genome were aligned to a completed reference genome using MAUVE [21,22]. Draft genomes were submitted to RAST [21,22] for annotation. Sequencing reads are available at NCBI SRA: TW16501, SRR5386013.

### 5.8. Incubation and Harvest of Lettuce for the Chlorine Survival Assay

Inoculated lettuce was prepared as described in Section 5.3 and used to determine if association with pre-harvest lettuce affected the pathogen’s ability to tolerate chlorine. For this experiment, the inoculated lettuce plants were incubated at 75% RH under June harvest conditions (14.8 h light, max temp 20 °C, min temp 12.2 °C). This experiment was replicated twice with two technical replicates per biological replicate for each strain. For each biological replicate, two technical replicates consisting of lettuce leaves from two pots were harvested on days 0, 1, 3, and 5 post-inoculation. For day 0 samples, inoculated lettuce leaves were harvested 1 h after spraying, allowing the surface of the leaves to dry completely.

### 5.9. Chlorine Survival Assay

Chlorinated water (50 ppm) was prepared by adding 1.125 mL of XY-12 (sodium hypochlorite, Ecolab 42016) in 1.8 L of sterile 0.05 M KH_2_PO_4_ at pH 6.8, similar to that described by Al-Nabulsi et al. [54]. The average pH of the solution upon addition of XY-12 was 6.93 ± 0.04. The concentration of available chlorine was determined by a chlorine testing kit (#322, Ecolab, St. Paul, MN, USA) and found to be 40 ppm. The chlorine solution was held chilled at 4 °C.

To determine if the length of time pathogens were associated with pre-harvest lettuce impacted their ability to resist chlorine decontamination, lettuce was inoculated with each pathogen and incubated under conditions described in Section 5.2 Leaves from two pots of lettuce (~15–20 g) were mixed in one sterile Whirl-Pak bag using sterile tweezers. The lettuce was weighed and approximately divided into half. To one bag, 500 mL of sterile 0.05 M KH_2_PO_4_ was added, while 500 mL of chlorine solution was added to the other bag. The bags were closed and gently swirled in a circular motion for 2 min. To the bags with chlorine solution, 0.3 mL of 0.5 M sodium thiosulfate was added, and the bag was gently shaken for 20 s. The pH of the chlorine solution following lettuce washing was an average of 6.88 ± 0.02. Once the neutralizing solution was added, the leaves were transferred to new bags for the enumeration of remaining pathogens.

### 5.10. Bacterial Enumeration

Cut lettuce leaves were placed in sterile plastic bags, weighed, and diluted 1:10 with PBS. Bags were homogenized in a laboratory homogenizer (IUL Instruments masticator, S.A, Barcelona, Spain) for 90 s. Cells were quantified by serially diluting the samples and plating in duplicate on MacConkey agar using an Autoplate 4000 (Spiral Biotech, Norwood, MA, USA). Plates were incubated for 24 h at 37 °C and colonies were counted using the Q count (Model 530, Spiral Biotech, Norwood, MA, USA).

### 5.11. Statistical Analysis

The experimental design consisted of 4 EHEC strains spray inoculated on greenhouse cultivated lettuce. Each experiment was replicated twice, and each replicate consisted of two technical replicates of inoculated lettuce. Microbial data (CFU/mL) were divided by individual lettuce weights and log-transformed (log CFU/g of lettuce) before statistical analysis. Mean and standard deviations were obtained from log cfu/g of lettuce for each harvest day. The log decrease in bacterial numbers on each day post-inoculation was calculated by calculating the difference between the counts on either day 1, 3, or 5 post-inoculation and on day 0 for each biological replicate. Statistically significant differences in survival were identified with the general linear model (GLM) procedure of the Statistical Analysis System (SAS v.9.3, Cary. NC, USA, 2017), using the following model: Log decrease = μ + RH + harvest season + RH x harvest season + serotype + biological replicate + technical replicate + error. Tukey’s test was used for comparisons, and an adjusted *p*-value <0.05 was considered significant.

For the chlorine survival assay, the average log CFU/g of lettuce recovered after chlorine wash was subtracted from average log CFU/g recovered after washing in buffered water. This difference in survival (termed “reduction attributable to chlorine”) was used to identify which strains exhibited enhanced resistance to chlorine with the day of harvest as the time factor. Tukey’s test was used for multiple comparisons, and an adjusted *p*-value < 0.05 was considered significant.

### 5.12. RNA-seq Data Analysis

Reads were mapped to their respective genomes using BWA-MEM [55]. SAMtools was used to determine the read count per open reading frame [56], which was used as input for analysis in BaySeq [57]. Differential expression (DE) of protein-coding genes was analyzed based on the total coverage obtained for each gene or feature. DE analyses were performed for each strain to determine (i) DE of genes on each day post-inoculation, (ii) DE of genes on day 1 vs. days 3 and 5, (iii) DE of genes on day 3 vs. days 1 and 5, and (iv) DE of genes on day 5 vs. days 1 and 3. For each strain, we created an NDE model for non-differentially expressed and a DE model for differentially expressed. Likelihoods that a gene or feature belongs to the DE model, and their respective false discovery rate (FDR) were estimated. Genes or features with FDR values <0.05 and fold change (FC) of either <0.65 or >1.5 (representing up- or down-regulation of >1.5-fold) were considered significantly differentially expressed.

## Figures and Tables

**Table 1:** Isolates used in this study.

Isolate	Pathogen	Serotype	Source	Year of Isolation
TW08264	EHEC	O157:H7	Japan sprouts outbreak (Sakai)	1996
TW014359	EHEC	O157:H7	US Spinach outbreak	2006
TW09184	EHEC	O26:H11	Human sporadic	2003
TW016501	EHEC	O26:H11	US Sprouts outbreak	2012

**Table 2:** Average log CFU/g lettuce of enterohemorrhagic *Escherichia coli* (EHEC) strains on pre-harvest lettuce plants by season and relative humidity (RH) level.

Season	RH (%)	Day	Average Log CFU/g Lettuce			
			Sakai O157	Spinach O157	TW09184 O26	Sprouts O26
March	45	0	7.0 ± 0.4	6.6 ± 0.1	7.0 ± 0.4	7.2 ± 0.2
		1	5.6 ± 0.2	4.5 ± 0.6	6.1 ± 0.3	6.0 ± 0.4
		3	5.1 ± 0.8	4.5 ± 0.4	5.6 ± 0.2	5.4 ± 0.4
		5	5.0 ± 0.5	4.0 ± 0.2	5.5 ± 0.4	5.3 ± 0.2
	75	0	6.7 ± 0.1	6.7 ± 0.6	7.2 ± 0.1	7.1 ± 0.1
		1	6.1 ± 0.3	5.3 ± 0.5	6.3 ± 0.3	6.2 ± 0.2
		3	5.8 ± 0.4	4.2 ± 0.5	5.7 ± 0.6	5.5 ± 0.7
		5	4.6 ± 0.9	3.7 ± 0.6	5.4 ± 0.2	5.1 ± 0.2
June	45	0	6.8 ± 0.4	6.8 ± 0.4	7.1 ± 0.1	7.0 ± 0.3
		1	6.0 ± 0.2	5.5 ± 0.1	6.4 ± 0.3	6.3 ± 0.4
		3	5.5 ± 0.1	4.2 ± 0.7	5.7 ± 0.3	6.2 ± 0.3
		5	4.8 ± 0.2	3.6 ± 0.5	5.0 ± 0.5	5.1 ± 0.3
	75	0	8.5 ± 0.2	8.1 ± 0.1	8.2 ± 0.5	8.3 ± 0.1
		1	6.7 ± 0.1	5.9 ± 0.6	7.0 ± 0.3	6.7 ± 0.5
		3	4.9 ± 0.9	4.0 ± 0.3	5.9 ± 0.4	6.1 ± 0.6
		5	4.9 ± 0.6	3.8 ± 0.8	4.6 ± 0.7	5.3 ± 0.5

**Table 3:** Differences in EHEC survival after chlorine wash over 5 days incubation on lettuce under June 75% RH conditions.

Strain	Log Difference in CFU/g Lettuce between Buffered Water Wash and Chlorine Wash			
	Day 0 *	Day 1	Day 3	Day 5
Sakai O157	0.94 ± 0.14 AB ^z^	1.03 ± 0.29 A ^z^	0.36 ± 0.17 A ^y^	0.34 ± 0.20 A ^y^
Spinach O157	0.67 ± 0.10 B ^z^	0.66 ± 0.28 A ^z^	0.17 ± 0.13 A ^y^	0.33 ± 0.19 A ^y^
TW09184 O26	1.13 ± 0.06 A ^z^	0.79 ± 0.25 A ^z^	0.35 ± 0.25 A ^y^	0.81 ± 0.25 A ^z^
Sprouts O26	0.64 ± 0.26 B ^z^	1.02 ± 0.30 A ^z^	0.09 ± 0.03 A ^y^	0.51 ± 0.30 A ^z^

* Significant differences within columns are indicated by capital letters, while significant differences across rows are represented by lowercase superscripted letters.

**Table 4:** Stress response and virulence genes significantly differentially expressed across 5-day incubation period on lettuce plants for O157 Sakai.

ORF ID	Gene	Function	Significant Differential Gene Expression (Fold Change)				
			d1/d3	d3/d1	d3/d5	d5/d1	d5/d3
ECs0025	*espX*	T3SS effector-like protein EspX		1.9		5.6	3.0
ECs0472	*espY3*	T3SS effector-like protein EspY		1.7		4.2	2.5
ECs0865	*ybhM*	BAX Inhibitor-1 family inner membrane protein				4.6	5.1
ECs1274	*grvA*	Transcriptional regulator		1.8		3.9	2.1
ECs1388	*pchD*	Putative transcriptional regulator				3.7	3.9
ECs1417	*csgD*	Transcriptional regulator CsgD				4.5	3.3
ECs1438	*bssS*	biofilm regulator				3.4	4.2
ECs1490	*bhsA*	multiple stress resistance protein (YcfR)				3.5	4.2
ECs1926	*zntB*	Zinc transport protein ZntB				1.8	1.8
ECs2062	*ybfL*	type IV secretion protein Rhs				3.9	3.0
ECs2155	*nleG6-2*	T3SS secreted effector NleG				4.0	3.3
ECs2291	*ynfC*	Hypothetical UPF0257 lipoprotein ynfC precursor				2.4	2.7
ECs2333	*blr*	beta-lactam resistance membrane protein		1.8		11.6	6.3
ECs2672	*espR3*	T3SS effector-like protein EspR				5.3	3.9
ECs2765	*dicC*	cell division control protein				4.9	4.1
ECs2844	*wzy*	O antigen polymerase		1.5		5.6	3.6
ECs3124	*glpQ*	Glycerophosphoryl diester phosphodiesterase				2.2	2.4
ECs3155	*elaA*	acetyltransferase				2.0	2.2
ECs3241	*lacY*	galactosidase permease				2.4	2.6
ECs3728	*eivJ1*	type III secretion system protein EivJ1		1.7		5.4	3.2
ECs3729	*eivI*	type III secretion apparatus protein EivI		1.5		5.7	3.8
ECs3855	*espL2*	T3SS secreted effector EspL				3.7	2.7
ECs3858	*nleE*	T3SS secreted effector NleE				5.2	4.9
ECs3907	*qseB*	Two-component system response regulator QseB				1.9	2.1
ECs4188	*hopD*	Leader peptidase (Prepilin peptidase)				2.4	2.7
ECs4366	*uspB*	Universal stress protein B				3.8	4.1
ECs4392	*gadE*	Transcriptional activator GadE	1.9			4.3	8.2
ECs4502	*waaR*	UDP-galactose:(galactosyl) galactosyltransferase		1.8		9.7	5.5
ECs4574	*sepD*	type III secretion system protein SepD		1.7		10.5	6.1
ECs4578	*grlR*	negative regulator GrlR				6.5	4.8
ECs4580	*escU*	Type III secretion inner membrane protein				6.6	5.9
ECs4584		Orf5—T3SS component		1.9		11.3	5.9
ECs4586		Orf3—T3SS component				5.0	4.4
ECs5048	*espX5*	T3SS effector-like protein EspX		1.9		3.6	1.9

**Table 5:** Stress response genes significantly differentially expressed on days 3 and 5 in O26 sprouts.

Homologous ORF in Sakai	Gene	Function	Significant Differential Gene Expression (Fold Change)	
			d3/d1	d5/d1
ECs_0014	*dnaK*	chaperone Hsp70	2.3	1.7
ECs_0439	*yaiA*	OxyR-regulated protein	2.7	2.2
ECs_0466	*nrdR*	transcriptional regulator NrdR	1.8	1.5
ECs_0489	*bolA*	transcriptional regulator BolA	2.1	2.0
ECs_0662	*cspE*	cold-shock protein CspE	4.2	1.9
ECs_0966	*cspD*	cold-shock protein CspD	1.6	
ECs_1041	*ompA*	outer membrane protein A	2.4	1.7
ECs_1154	*cbpM*	chaperone modulatory protein CbpM	1.8	1.8
ECs_1387	*ybdM*	transcriptional regulator	2.1	1.7
ECs_1438	*bssS*	transcriptional regulator biofilm	2.5	1.6
ECs_1683	*ycgB*	SpoVR family stationary phase protein	1.7	1.6
ECs_1883	*pspC*	envelope stress response membrane protein PspC	4.0	2.5
ECs_1885	*pspE*	thiosulfate sulfurtransferase PspE	5.5	2.8
ECs_1915	*fnr*	transcriptional regulator FNR	1.6	1.6
ECs_2084	*sra*	stationary-phase-induced ribosome-associated protein	3.1	2.4
ECs_2086	*osmC*	peroxiredoxin OsmC	2.8	1.9
ECs_2145	*ydeI*	hydrogen peroxide resistance OB fold protein	2.2	1.7
ECs_2355	*sodC*	superoxide dismutase [Cu-Zn] SodC2	3.4	2.9
ECs_2504	*yeaQ*	stress response membrane protein	2.7	2.7
ECs_2558	*yebG*	damage-inducible protein YebG	2.3	1.8
ECs_3271	*mntH*	manganese/divalent cation transporter	1.5	
ECs_3476	*grpE*	molecular chaperone GrpE	1.8	
ECs_3553	*csrA*	carbon storage regulator	2.4	1.4
ECs_3556	*recA*	DNA recombination/repair protein RecA	1.7	1.6
ECs_3595	*rpoS*	RNA polymerase sigma factor RpoS	3.2	1.9
ECs_3887	*yghA*	NAD(P)-dependent oxidoreductase	1.9	1.7
ECs_4050	*nusA*	transcription elongation factor NusA	1.7	1.5
ECs_4390	*hdeA*	acid-resistance protein HdeA	2.6	2.0
ECs_4396	*gadX*	GAD regulon transcriptional activator	1.8	
ECs_4778	*hemG*	protoporphyrinogen oxidase	1.6	1.4
ECs_4789	*hemN*	coproporphyrinogen III oxidase		1.5
ECs_4923	*hupA*	transcriptional regulator HU subunit alpha	2.3	1.9
ECs_5029	*zur*	transcriptional regulator Zur	2.3	2.0
ECs_5039	*yjbR*	MmcQ/YjbR family DNA-binding protein	1.5	
ECs_5123	*groES*	co-chaperonin GroES	2.5	2.2

## Supplementary Materials

The following are available online at https://www.mdpi.com/2072-6651/11/11/675/s1, Table S1: Significantly differentially expressed genes in O157 Sakai over time of incubation on pre-harvest lettuce. Table S2: Significantly differentially expressed genes in O26 sprouts over time of incubation on pre-harvest lettuce.
