# Supplementary material for: Pre-Harvest Survival and Post-Harvest Chlorine Tolerance of Enterohemorrhagic Escherichia coli on Lettuce
Source: Toxins (Basel). 2019 Nov 19;11(11):675. doi: 10.3390/toxins11110675 (PMC6891304; doi:10.3390/toxins11110675)
Supplement: Supplementary file 1 [file toxins-11-00675-s001.pdf]

Supplementary Materials: Pre-Harvest Survival and Post-Harvest Chlorine Tolerance of Enterohemorrhagic *Escherichia coli* on Lettuce

Deepti Tyagi, Autumn L. Kraft, Sara Levadney Smith, Sherry E. Roof, Julie S. Sherwood, Martin Wiedmann and Teresa M. Bergholz

Table S1. Significantly differentially expressed genes in O157 Sakai over time associated with pre-harvest lettuce.

ORF ID	Gene	Function	Significant Differential Gene Expression (Fold Change)				
			d1/d3	d3/d1	d3/d5	d5/d1	d5/d3
ECs0023		Uncharacterized fimbrial chaperone YehC precursor				6.4	5.7
ECs0025	*espX*	T3SS effector-like protein EspX		1.9		5.6	3
ECs0212		hypothetical protein		2.5		9.3	3.8
ECs0213		hypothetical protein		1.8		6.8	3.7
ECs0216		type VI secretion system effector Hcp1 family protein				3.5	2.4
ECs0240		hypothetical protein				11.3	9.8
ECs0295		hypothetical protein		1.9		10.2	5.4
ECs0304		hypothetical protein				4.9	3.9
ECs0307		hypothetical membrane protein		2		3.8	1.9
ECs0348		hypothetical protein		1.6		5.6	3.5
ECs0382	*yahN*	Threonine efflux protein				5.7	4.9
ECs0472	*espY3*	T3SS effector-like protein EspY		1.7		4.2	2.5
ECs0548		hypothetical adhesin protein		1.7		5.4	3.2
ECs0549		hypothetical protein				3.2	3.3
ECs0561	*ybbD*	hypothetical protein		2.7		4	1.5
ECs0604		hypothetical protein				3.9	4.2
ECs0730	*ybfC*	hypothetical protein				4.3	3.8
ECs0865	*ybhM*	BAX Inhibitor-1 family inner membrane protein				4.6	5.1
ECs1099		hypothetical protein				3.7	3.2
ECs1192		hypothetical protein				3.1	3.1
ECs1274	*grvA*	Transcriptional regulator		1.8		3.9	2.1
ECs1316		Diacylglycerol kinase				3.7	3
ECs1374		putative membrane protein		1.9		5	2.7
ECs1388	*pchD*	Putative transcriptional regulator				3.7	3.9
ECs1417	*csgD*	Transcriptional regulator CsgD				4.5	3.3
ECs1438	*bssS*	biofilm regulator				3.4	4.2

ECs1490	*bhsA*	multiple stress resistance protein (YcfR)				3.5	4.2
ECs1581		hypothetical protein				5.3	3.7
ECs1585		hypothetical protein				3.4	3.9
ECs1654	*yciF*	hypothetical protein				3.4	3.4
ECs1926	*zntB*	Zinc transport protein ZntB				1.8	1.8
ECs1955		hypothetical protein		1.5		6.1	4.1
ECs2017		hypothetical protein		2.8		7.3	2.6
ECs2018		hypothetical protein				5.6	3.9
ECs2062	*ybfL*	type IV secretion protein Rhs				3.9	3
ECs2155	*nleG6-2*	T3SS secreted effector NleG				4	3.3
ECs2230		hypothetical protein	1.7			2.4	4.1
ECs2270		hypothetical protein	2.3			3	6.9
ECs2291	*ynfC*	Hypothetical UPF0257 lipoprotein ynfC precursor				2.4	2.7
ECs2333	*blr*	beta-lactam resistance membrane protein		1.8		11.6	6.3
ECs2436	*ydjO*	hypothetical protein		2.5		5.6	2.3
ECs2672	*espR3*	T3SS effector-like protein EspR				5.3	3.9
ECs2743		putative holin protein				2.3	2.7
ECs2755		hypothetical protein	2.1			4.1	8.5
ECs2765	*dicC*	cell division control protein				4.9	4.1
ECs2799		hypothetical protein				4.6	3.9
ECs2844	*wzy*	O antigen polymerase		1.5		5.6	3.6
ECs2897	*gatA*	PTS system galactitol-specific IIA component		1.8	1.7		
ECs2910		putative outer membrane protein		1.6		3.8	2.4
ECs2984		hypothetical protein				3.6	2.8
ECs2991		hypothetical protein		3		3.8	1.3
ECs3124	*glpQ*	Glycerophosphoryl diester phosphodiesterase				2.2	2.4
ECs3155	*elaA*	acetyltransferase				2	2.2
ECs3241	*lacY*	galactosidase permease				2.4	2.6
ECs3355	*yfgO*	Putative permease PerM (YfgO)				2.3	2.5
ECs3510		DNA-binding protein		1.8		4.3	2.4
ECs3513		putative phage protein				4.2	3.5
ECs3517	*ypjB*	hypothetical protein		1.7		7.5	4.3
ECs3714		hypothetical protein		1.6		18	11
ECs3728	*eivJ1*	type III secretion system protein EivJ1		1.7		5.4	3.2
ECs3729	*eivI*	type III secretion apparatus protein EivI		1.5		5.7	3.8

ECs3760	*uacT*	Xanthine permease				1.9	1.9
ECs3855	*espL2*	T3SS secreted effector EspL				3.7	2.7
ECs3858	*nleE*	T3SS secreted effector NleE				5.2	4.9
ECs3907	*qseB*	Two-component system response regulator QseB				1.9	2.1
ECs4001	*yhaC*	hypothetical protein		1.6		5.8	3.7
ECs4188	*hopD*	Leader peptidase (Prepilin peptidase)				2.4	2.7
ECs4291		hypothetical protein		1.7		4	2.4
ECs4292		hypothetical protein				10.1	7.3
ECs4316	*yhhM*	Putative receptor		1.5	1.5		
ECs4362	*yheL*	hypothetical protein		1.6		4.5	2.8
ECs4366	*uspB*	Universal stress protein B				3.8	4.1
ECs4392	*gadE*	Transcriptional activator GadE	1.9			4.3	8.2
ECs4471	*yibG*	hypothetical protein				3.9	3.5
ECs4502	*waaR*	UDP-galactose:(galactosyl) galactosyltransferase		1.8		9.7	5.5
ECs4574	*sepD*	type III secretion system protein SepD		1.7		10.5	6.1
ECs4578	*grlR*	negative regulator GrlR				6.5	4.8
ECs4580	*escU*	Type III secretion inner membrane protein				6.6	5.9
ECs4584		Orf5 - T3SS component		1.9		11.3	5.9
ECs4586		Orf3 - T3SS component				5	4.4
ECs4607		hypothetical protein				7.1	6.3
ECs4747		hypothetical protein		1.9		6	3.1
ECs4748		membrane protein				6	7.8
ECs4846	*yiiR*	Putative membrane protein	1.6			4.6	7.4
ECs4902		hypothetical protein		2.6	2.8		
ECs5048	*espX5*	T3SS effector-like protein EspX		1.9		3.6	1.9
ECs5160	*yjfL*	Membrane protein with DUF350 domain		2		4.5	2.2
ECs5184	*ytfB*	Putative cell envelope opacity-associated protein A				2	2
ECs5193	*ytfI*	hypothetical protein				3.5	2.9
ECs5230	*yjgL*	SopA-like hexapeptide repeat protein				5.8	4.1

Table S2. Significantly differentially expressed genes in O26 sprouts over time associated with pre-harvest lettuce.

ORF ID in RM8426 [a]	Homologous ORF in Sakai [b]	Gene	Function	Significant Differential Gene Expression (Fold Change)			
				d3/d1	d3/d5	d5/d1	d5/d3
I3M_00590	ECs_0014	*dnaK*	chaperone Hsp70	2.3		1.7	
I3M_01205	ECs_0131	*yadG*	inner membrane protein			1.5	1.6
I3M_01540	ECs_0192	*yaeQ*	hypothetical protein			1.6	
I3M_03960	ECs_0241	*ydcC*	ISAs1 family transposase	2			
I3M_01795	ECs_0249	*gmhA*	phosphoheptose isomerase	1.8			
I3M_02495	ECs_0385	*prpB*	methylisocitrate lyase	2.6		1.8	
I3M_02500	ECs_0386	*prpC*	2-methylcitrate synthase	1.9		1.8	
I3M_02780	ECs_0439	*yaiA*	OxyR-regulated protein	2.7		2.2	
I3M_02905	ECs_0466	*nrdR*	transcriptional regulator NrdR	1.8		1.5	
I3M_03030	ECs_0489	*bolA*	transcriptional regulator BolA	2.1		2	
I3M_03070	ECs_0496	*ybaV*	hypothetical protein			1.5	
I3M_03255	ECs_0527	*adk*	adenylate kinase	1.5			
I3M_04310	ECs_0662	*cspE*	cold-shock protein CspE	4.2		1.9	
I3M_04325	ECs_0665	*tatE*	TatABCE protein translocation system subunit	2.4		1.5	
I3M_04355	ECs_0669	*ybeD*	hypothetical protein	3.7		2.3	
I3M_04990	ECs_0783	*gpmA*	2,3-bisphosphoglycerate-dependent phosphoglycerate mutase	1.8		1.6	
I3M_03735	ECs_0819	*rrrD*	phage endolysin	1.6			
I3M_12435	ECs_0836		phage tail assembly protein T	1.7		2	
I3M_05540	ECs_0860	*moaB*	molybdenum cofactor biosynthesis protein	1.7		2	
I3M_05850	ECs_0917	*yliI*	PQQ-dependent sugar dehydrogenase	1.5			
I3M_06075	ECs_0966	*cspD*	cold-shock protein CspD	1.6			
I3M_06280	ECs_1003	*elyC*	envelope biogenesis factor	1.6		1.6	
I3M_06485	ECs_1041	*ompA*	outer membrane protein A	2.4		1.7	
I3M_06510	ECs_1045	*yccF*	inner membrane protein	1.7		1.5	
I3M_12640	ECs_1064		hypothetical protein			1.8	
I3M_09075	ECs_1154	*cbpM*	chaperone modulatory protein CbpM	1.8		1.8	
I3M_07515	ECs_1312	*traT*	complement resistance protein TraT	1.7			
I3M_07565	ECs_1322	*ureA*	urease subunit gamma			2.3	
I3M_07880	ECs_1387	*ybdM*	transcriptional regulator	2.1		1.7	
I3M_30535	ECs_1402		antirestriction protein	1.6		1.2	
I3M_30555	ECs_1406	*yeeV*	hypothetical protein			1.5	1.6

I3M_08200	ECs_1438	*bssS*	transcriptional regulator biofilm	2.5	1.6
I3M_08320	ECs_1461	*flgL*	flagellar hook-filament junction protein FlgL	1.5	
I3M_08775	ECs_1540		HNH endonuclease	1.9	2.5
I3M_09825	ECs_1672	*pliG*	inhibitor of g-type lysozyme		1.9
I3M_09880	ECs_1683	*ycgB*	SpoVR family stationary phase protein	1.7	1.6
I3M_10080	ECs_1722	*chaB*	cation transport regulator	1.8	
I3M_10555	ECs_1829	*yciE*	ferritin-like domain-containing protein	2.1	1.6
I3M_10560	ECs_1830	*yciF*	YciE/YciF family protein	3.2	2.3
I3M_10840	ECs_1883	*pspC*	envelope stress response membrane protein PspC	4	2.5
I3M_10850	ECs_1885	*pspE*	thiosulfate sulfurtransferase PspE	5.5	2.8
I3M_10985	ECs_1915	*fnr*	transcriptional regulator FNR	1.6	1.6
I3M_11070	ECs_1933	*recT*	recombination protein RecT	1.5	
I3M_11575	ECs_2013	*ynbD*	hypothetical protein		1.9
I3M_11960	ECs_2084	*sra*	stationary-phase-induced ribosome-associated protein	3.1	2.4
I3M_11975	ECs_2086	*osmC*	peroxiredoxin OsmC	2.8	1.9
I3M_12325	ECs_2145	*ydeI*	hydrogen peroxide resistance OB fold protein	2.2	1.7
I3M_13020	ECs_2210		hypothetical protein	1.5	1.8
I3M_15105	ECs_2279		hypothetical protein	2.6	1.9
I3M_13100	ECs_2291	*ynfC*	YnfC family lipoprotein	1.5	1.5
I3M_13505	ECs_2344	*gst*	glutathione S-transferase	1.7	1.7
I3M_13560	ECs_2355	*sodC*	superoxide dismutase [Cu-Zn] SodC2	3.4	2.9
I3M_13765	ECs_2392	*ydiH*	hypothetical protein	2.5	
I3M_14175	ECs_2424	*rpmI*	50S ribosomal protein L35	2.4	
I3M_14470	ECs_2488	*gapA*	glyceraldehyde-3-phosphate dehydrogenase A	1.7	
I3M_14490	ECs_2492	*yeaG*	PrkA family serine protein kinase	1.7	1.8
I3M_14560	ECs_2504	*yeaQ*	GlsB/YeaQ/YmgE family stress response membrane protein	2.7	2.7
I3M_14805	ECs_2546	*yebV*	hypothetical protein	2.9	1.6
I3M_15205	ECs_2556	*yebE*	inner membrane protein	2.3	1.5
I3M_15210	ECs_2557	*yebF*	extracellular Colicin M immunity family protein	1.6	
I3M_15215	ECs_2558	*yebG*	damage-inducible protein YebG	2.3	1.8
I3M_15525	ECs_2604	*otsA*	trehalose-6-phosphate synthase	2	1.6
I3M_15530	ECs_2605	*otsB*	trehalose-6-phosphate phosphatase	3.1	2.6
I3M_15805	ECs_2691	*dsrB*	dsrB protein	2.4	2
I3M_15835	ECs_2695	*yodC*	hypothetical protein	2.9	2.3
I3M_16300	ECs_2811	*sbmC*	DNA gyrase inhibitor SbmC		1.7
I3M_16760	ECs_2900	*fbaB*	fructose-bisphosphate aldolase	1.6	1.5

I3M_17465	ECs_3097	*napF*	ferredoxin-type protein NapF	1.8	1.9
I3M_18115	ECs_3154	*elaB*	transmembrane protein	2.6	1.8
I3M_18120	ECs_3155	*elaA*	acetyltransferase	2	1.9
I3M_18290	ECs_3187	*folX*	dihydroneopterin triphosphate 2'-epimerase	1.6	
I3M_18980	ECs_3241		sucrose transport protein	1.5	1.8
I3M_19070	ECs_3258	*lpxP*	lipid A biosynthesis palmitoleoyltransferase	2	
3M_19140	ECs_3271	*mntH*	manganese/divalent cation transporter	1.5	
I3M_19270	ECs_3289	*crr*	glucose-specific enzyme IIA component of PTS	1.5	1.5
I3M_19350	ECs_3305	*ypeA*	acetyltransferase YpeA		1.7
I3M_19465	ECs_3327	*tktB*	transketolase	1.5	1.6
I3M_19475	ECs_3329	*nudK*	GDP-mannose pyrophosphatase NudK	1.6	1.7
I3M_19555	ECs_3340	*dapA*	4-hydroxy-tetrahydrodipicolinate synthase	1.6	
I3M_19835	ECs_3395	*iscU*	iron-sulfur cluster scaffold-like protein	2.2	
I3M_20080	ECs_3439	*rpoE*	ECF RNA polymerase sigma-E factor	2.8	2.5
I3M_20295	ECs_3476	*grpE*	molecular chaperone GrpE	1.8	
I3M_20360	ECs_3489		phage tail protein	1.9	1.8
I3M_20750	ECs_3526		peptidoglycan-binding protein LysM	2.6	1.7
I3M_20855	ECs_3549	*luxS*	S-ribosylhomocysteine lyase	1.6	
I3M_20900	ECs_3553	*csrA*	carbon storage regulator	2.4	1.4
I3M_20915	ECs_3556	*recA*	DNA recombination/repair protein RecA	1.7	1.6
I3M_21160	ECs_3595	*rpoS*	RNA polymerase sigma factor RpoS	3.2	1.9
I3M_21165	Ecs_3596	*nlpD*	murein hydrolase activator NlpD	3.7	2
I3M_21515	ECs_3669	*ygdI*	YgdI/YgdR family lipoprotein	3.7	2.7
I3M_22120	ECs_3771	*yqfA*	hemolysin III family protein	2	
I3M_22395	ECs_3812	*speB*	agmatinase	1.5	1.7
I3M_22820	ECs_3887	*yghA*	NAD(P)-dependent oxidoreductase	1.9	1.7
I3M_23025	ECs_3931	*glgS*	glycogen synthase	2.6	2.2
I3M_23185	ECs_3955	*patA*	putrescine:2-oxoglutaric acid aminotransferase	1.7	2.1
I3M_23335	ECs_3979	*yqjC*	DUF1090 domain-containing protein	2.2	1.5
I3M_23340	ECs_3980	*yqjD*		2.1	1.8
I3M_23725	ECs_4050	*nusA*	transcription elongation factor NusA	1.7	1.5
I3M_23775	ECs_4058	*rlmE*	23S rRNA (uridine(2552)-2'-O)-methyltransferase	1.9	1.5
I3M_24065	ECs_4111	*yhcN*	cadmium and peroxide resistance protein	2.7	
I3M_24070	ECs_4112	*yhcO*	barnase inhibitor		2.1
I3M_24345	ECs_4145		gamma carbonic anhydrase family protein	1.7	1.6
I3M_24370	ECs_4150	*smg*	hypothetical protein	1.7	

I3M_24415	ECs_4158	*yhdN*	hypothetical protein	1.9	1.5	
I3M_24450	ECs_4165	*secY*	preprotein translocase membrane subunit SecY	2.2		
I3M_24455	ECs_4166	*rplO*	50S ribosomal protein L15	2.1		
I3M_24475	ECs_4170	*rplF*	50S ribosomal protein L6	2.6		
I3M_24490	ECs_4173	*rplE*	50S ribosomal protein L5	2.3		
I3M_24565	ECs_4188	*hopD*	prepilin peptidase	1.7	1.6	
I3M_24570	ECs_4189	*bfr*	bacterioferritin	2.2	1.7	
I3M_25000	ECs_4256	*nfuA*	Fe-S biogenesis protein NfuA	1.7		
I3M_25285	ECs_4319	*tusA*	sulfurtransferase TusA		1.6	
I3M_25515	ECs_4390	*hdeA*	acid-resistance protein HdeA	2.6	2	
I3M_25555	ECs_4396	*gadX*	acid resistance regulon transcriptional activator	1.8		
I3M_25600	ECs_4405	*yhjH*	cyclic di-GMP phosphodiesterase YhjH		1.6	
I3M_28045	ECs_4440	*yiaG*	HTH_CROC1 family transcriptional regulator	2.8	1.5	
I3M_27700	ECs_4491	*envC*	murein hydrolase activator	1.7	2.1	
I3M_26990	ECs_4663	*pstC*	phosphate ABC transporter permease		1.6	
I3M_26885	ECs_4685	*asnC*	transcriptional activator of asnA		1.7	1.6
I3M_26325	ECs_4778	*hemG*	protoporphyrinogen oxidase	1.6	1.4	
I3M_26230	ECs_4789	*hemN*	coproporphyrinogen III oxidase		1.5	
I3M_26085	ECs_4815	*yiiE*	CopG family transcriptional regulator	2.6	2.1	
I3M_25845	ECs_4859	*hslV*	HslU--HslV peptidase proteolytic subunit	1.8		
I3M_25850	Ecs_4860	*hslU*	HslU--HslV peptidase ATPase subunit	1.8	1.6	
I3M_28440	ECs_4923	*hupA*	transcriptional regulator HU subunit alpha	2.3	1.9	
I3M_28505	ECs_4930	*yjaB*	acetyltransferase	1.7	1.7	
I3M_28515	ECs_4932	*aceB*	malate synthase A	2	1.4	
I3M_28760	ECs_5029	*zur*	transcriptional regulator Zur	2.3	2	
I3M_28820	ECs_5039	*yjbR*	MmcQ/YjbR family DNA-binding protein	1.5		
I3M_28945	ECs_5057	*nrfF*	heme lyase NrfEFG subunit NrfF	2	2.2	
I3M_29585	ECs_5123	*groES*	co-chaperonin GroES	2.5	2.2	
I3M_29755	ECs_5147	*miaA*	delta(2)-isopentenylpyrophosphate tRNA-adenosine transferase	2.1	1.8	
I3M_29760	ECs_5148	*hfq*	RNA-binding protein Hfq	2.6	1.9	
I3M_29780	ECs_5152	*yjeT*	DUF2065 domain-containing protein	1.8	1.9	
I3M_30050	ECs_5202	*chpS*	antitoxin ChpS	1.7	1.9	
I3M_31140	ECs_5352	*yjjX*	non-canonical purine NTP phosphatase	2	1.9	
I3M_11185	ECs_5710		hypothetical protein	3.3	2.7	
I3M_26680	ECs_5764		hypothetical protein	2.1	2.3	
I3M_31035	ECs_5780	*ytjA*	DUF1328 domain-containing protein	3.9	2.7	

I3M_05735	*mntS*	manganase accumulation protein MntS	1.7	
I3M_00890	*setA*	Putative transport protein		1.9
I3M_23180		hypothetical protein		1.6
I3M_16350	*yefM*	antitoxin YefM		1.5
I3M_03720		antiterminator protein Q	1.5	1.5
I3M_14080		phage tail protein I	1.6	1.8
I3M_24900	*igaA*	intracellular growth attenuator protein IgaA	1.6	1.5
I3M_26100		toxin HigB-2	1.7	1.5
I3M_12960		type II toxin-antitoxin system RelE/ParE family toxin	1.8	
I3M_19540	*vapB*	antitoxin	2	1.5
I3M_07035		hypothetical protein	2	
I3M_12575		hypothetical protein	2.2	3
I3M_01935	*flgB*	flagellar basal body rod protein FlgB	2.2	2
I3M_07805		hypothetical protein	2.4	2
I3M_00350		hypothetical protein on plasmid	2.6	2.1
I3M_16110		hypothetical protein	2.8	2.6
I3M_11190		hypothetical protein	5.2	3.5
I3M_25455		hypothetical protein	5.8	3.6
I3M_12905		hypothetical protein	1.8	2.2

[a] O26 sprouts ORFs mapped to ORFs in a completed *E. coli* O26 genome, from strain RM8426 (CP028116.1). [b] O26 sprouts ORFS mapped to homologous ORFs in O157 Sakai.
